# Supplementary material for: High expression of Helicobacter pylori VapD in both the intracellular environment and biopsies from gastric patients with severity
Source: PLoS One. 2020 Mar 12;15(3):e0230220. doi: 10.1371/journal.pone.0230220 (PMC7067408; doi:10.1371/journal.pone.0230220)
Supplement: S5 Raw images — (PDF) [file pone.0230220.s006.pdf]

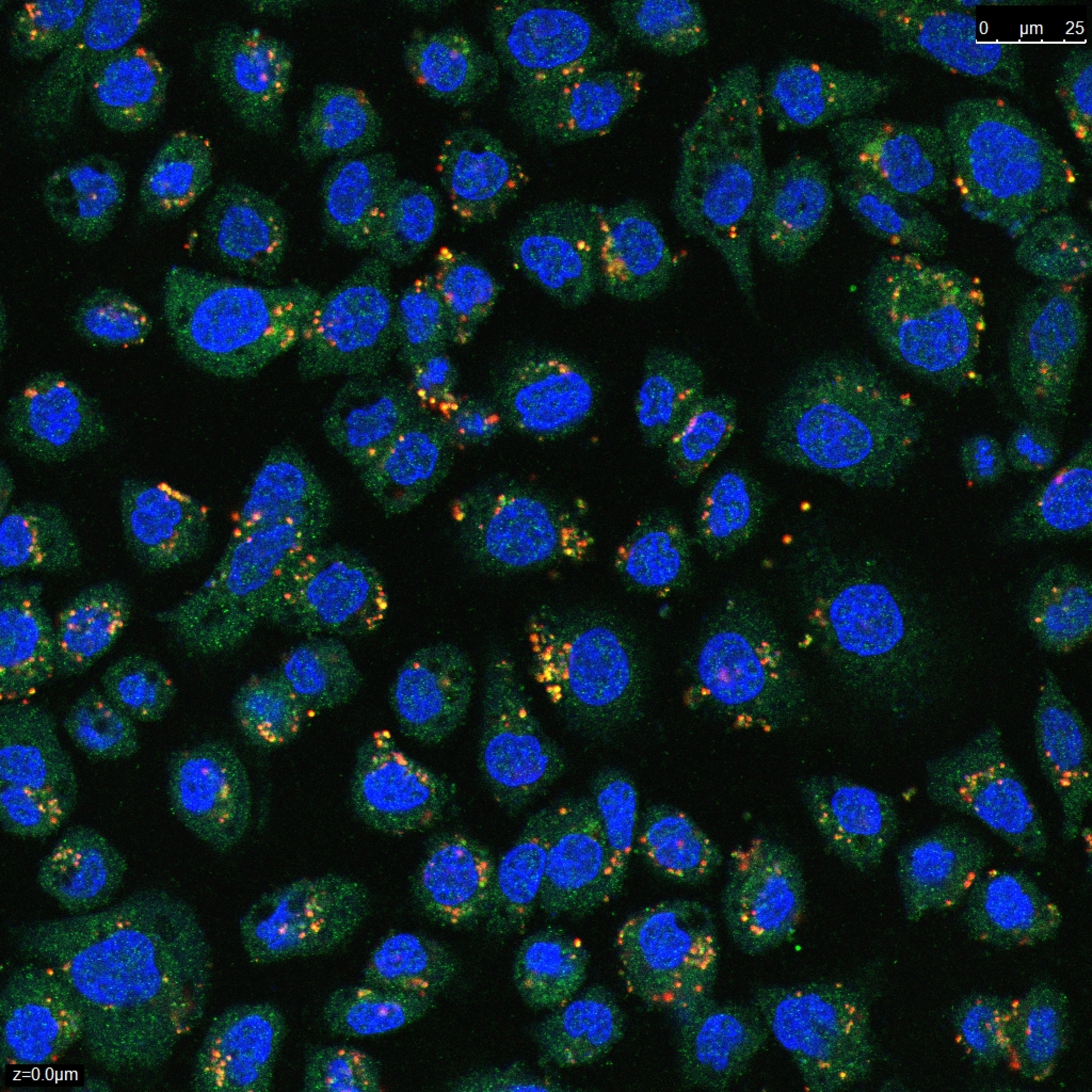
0 µm 25
z=0.0µm

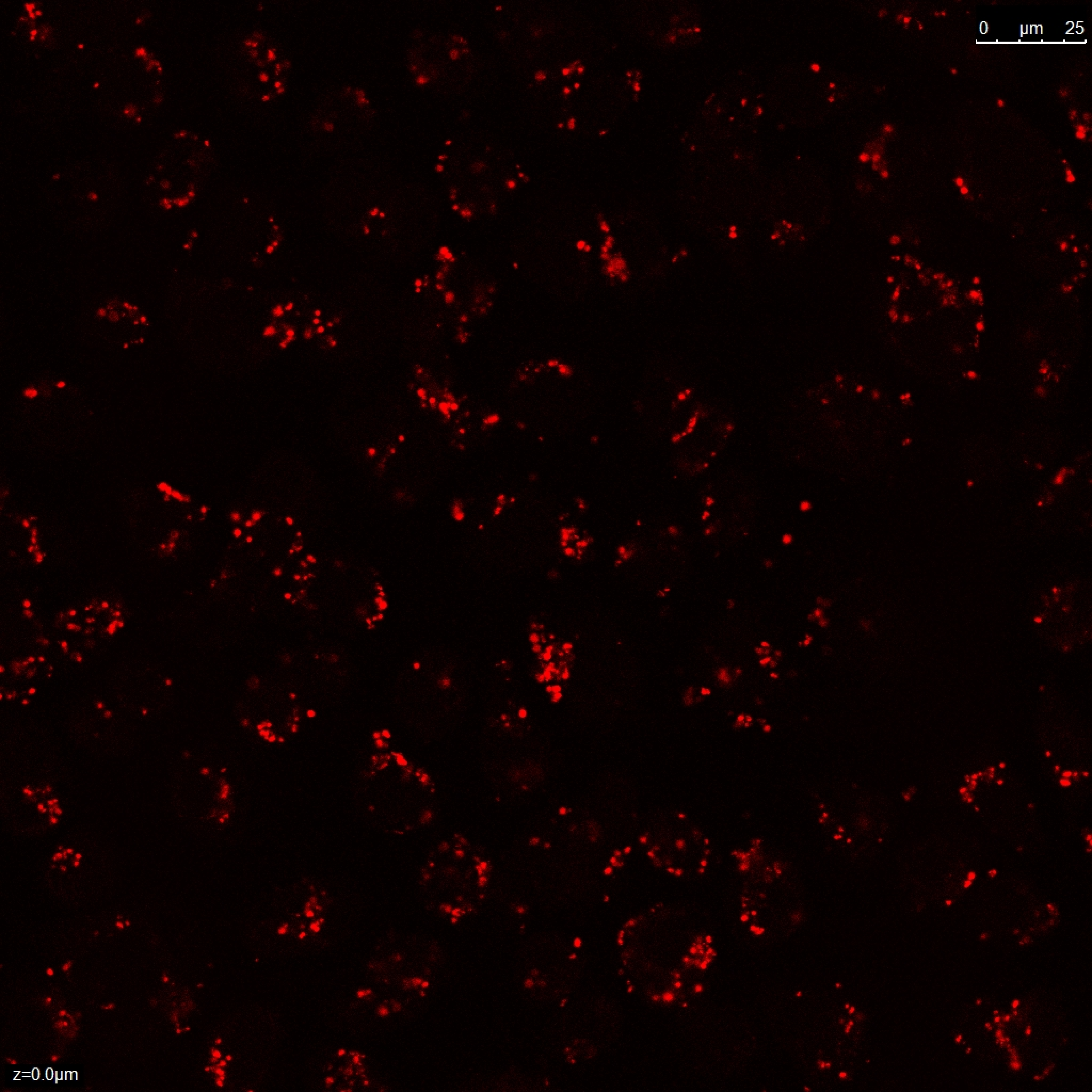
0 μm 25
z=0.0μm

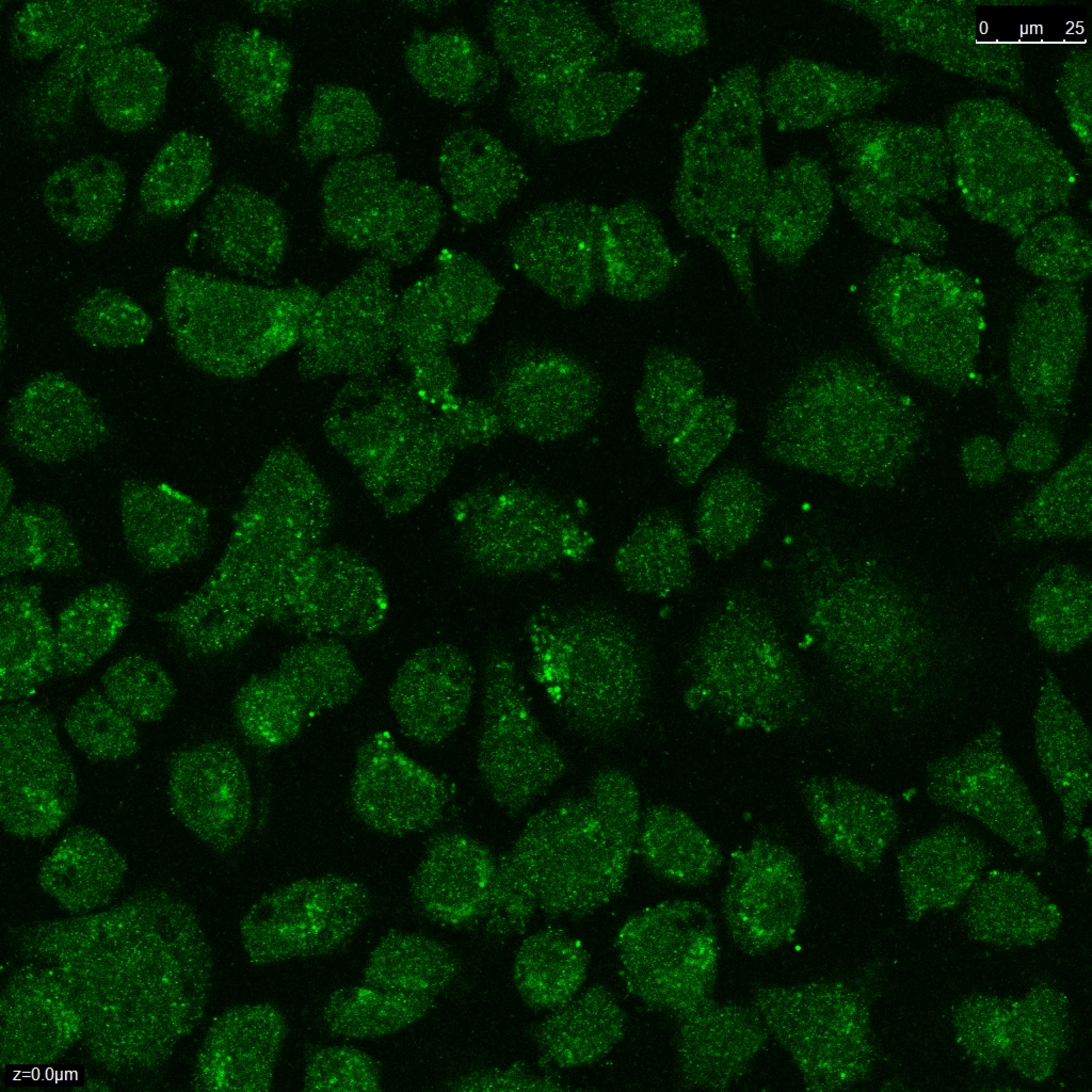
0 μm 25
z=0.0μm

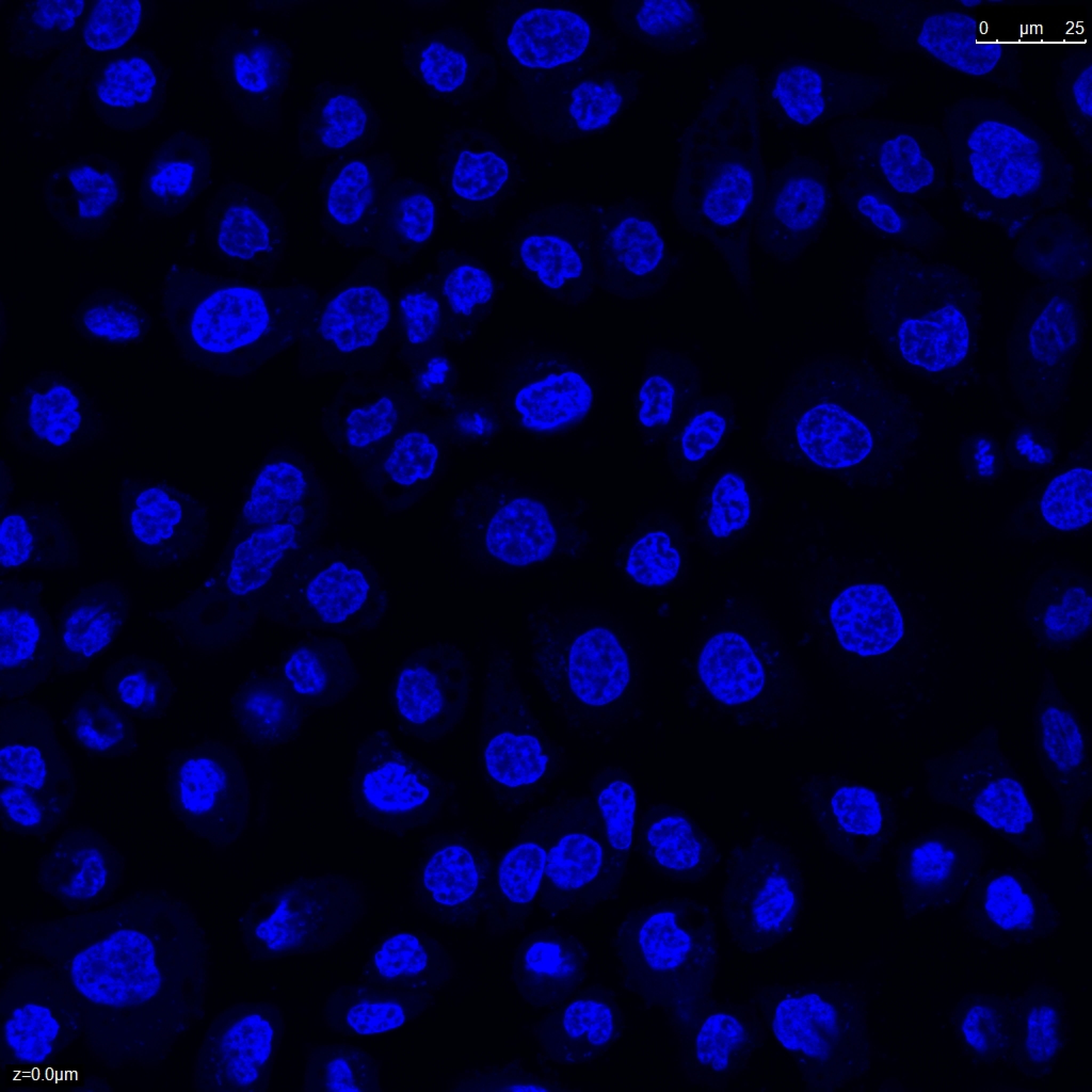
0 µm 25
z=0.0µm

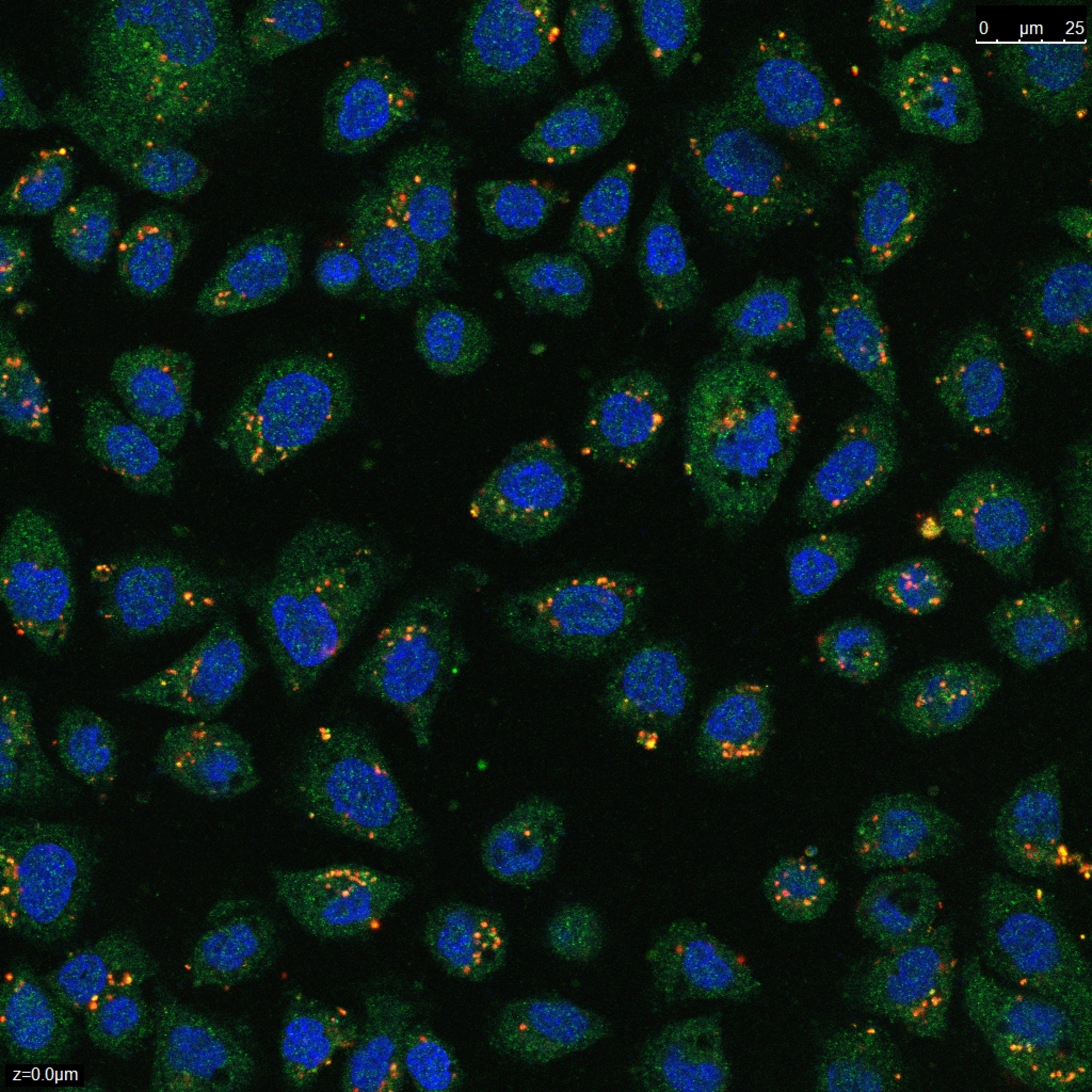
0 µm 25
z=0.0µm

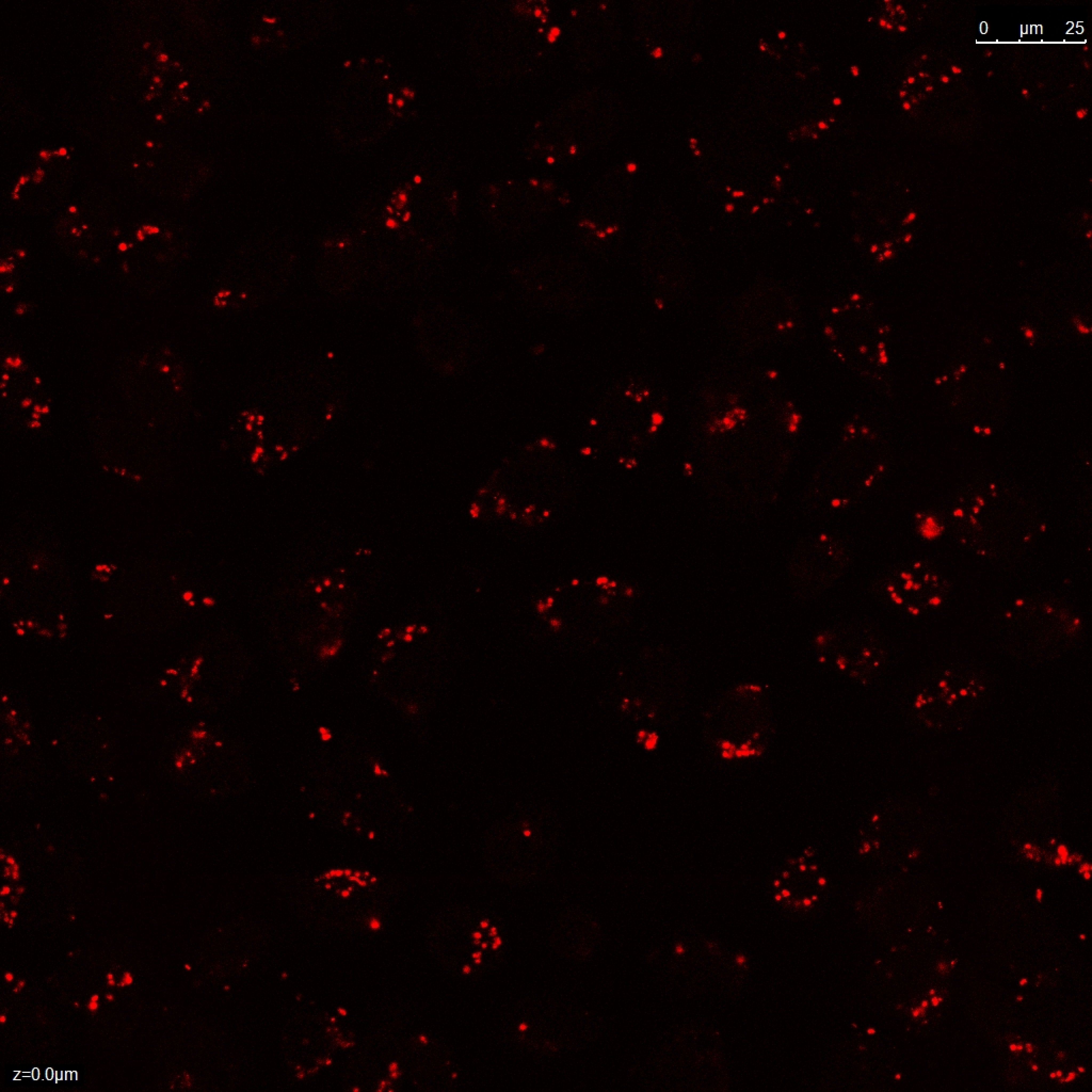
0 µm 25
z=0.0µm

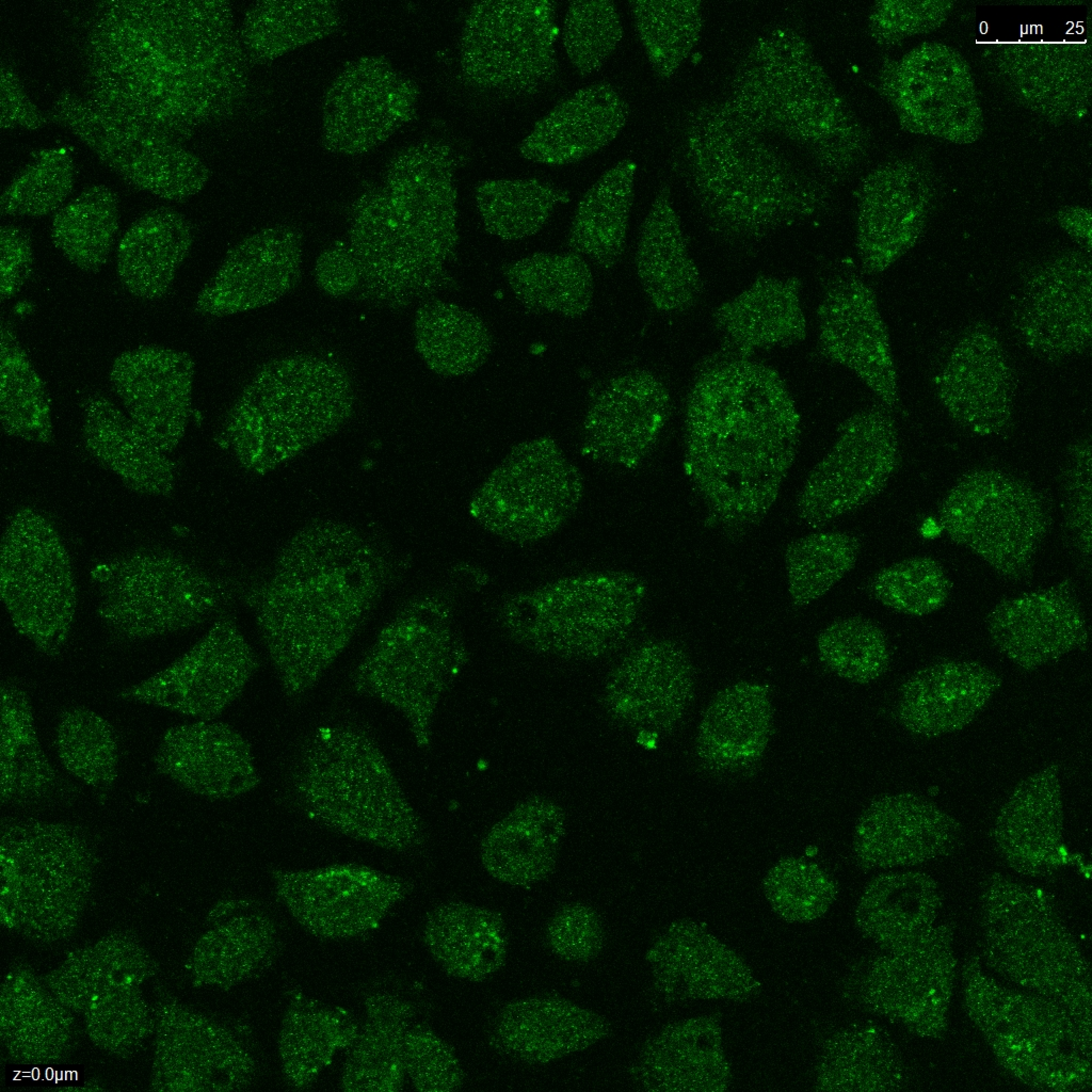
0 μm 25
z=0.0μm

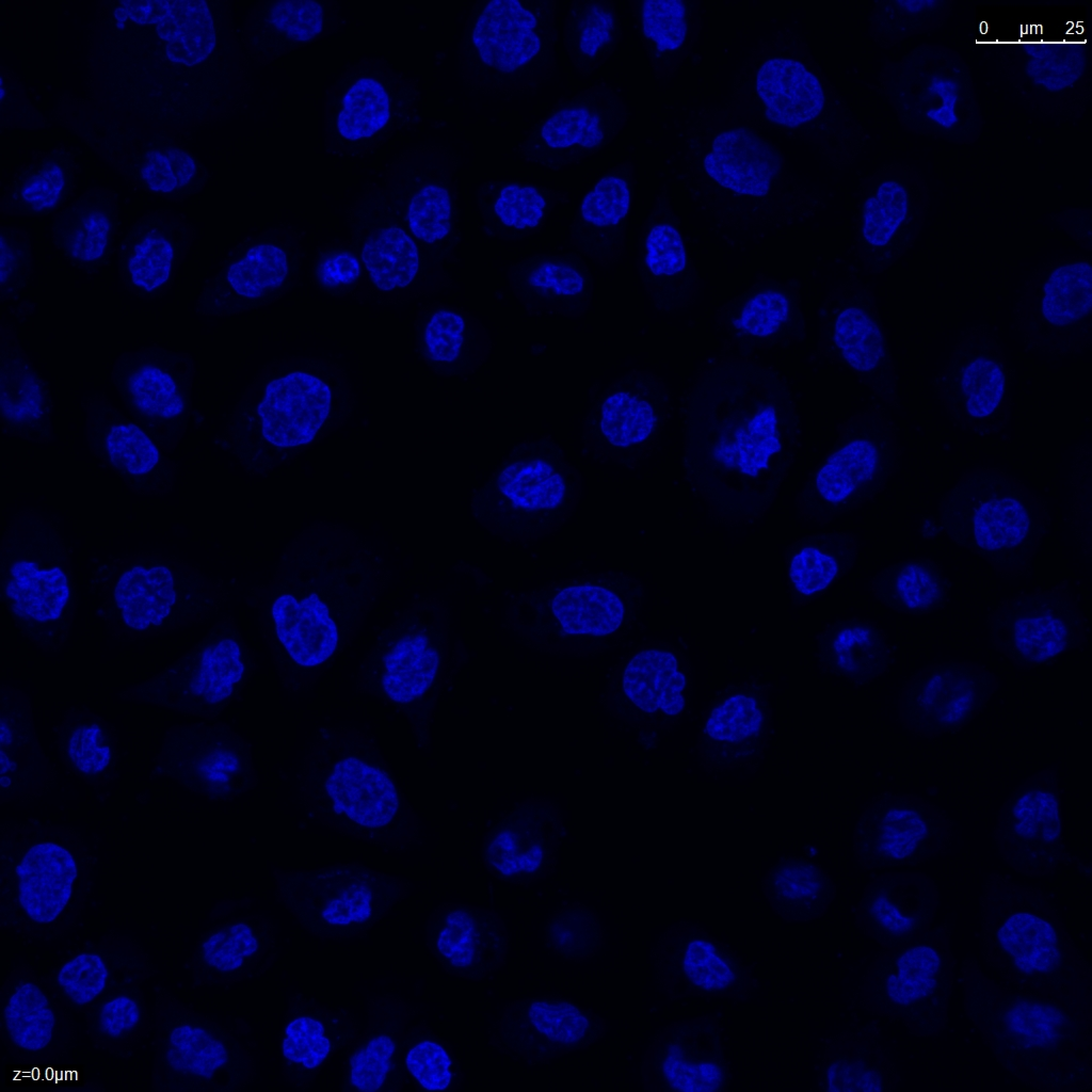
0 µm 25
z=0.0µm

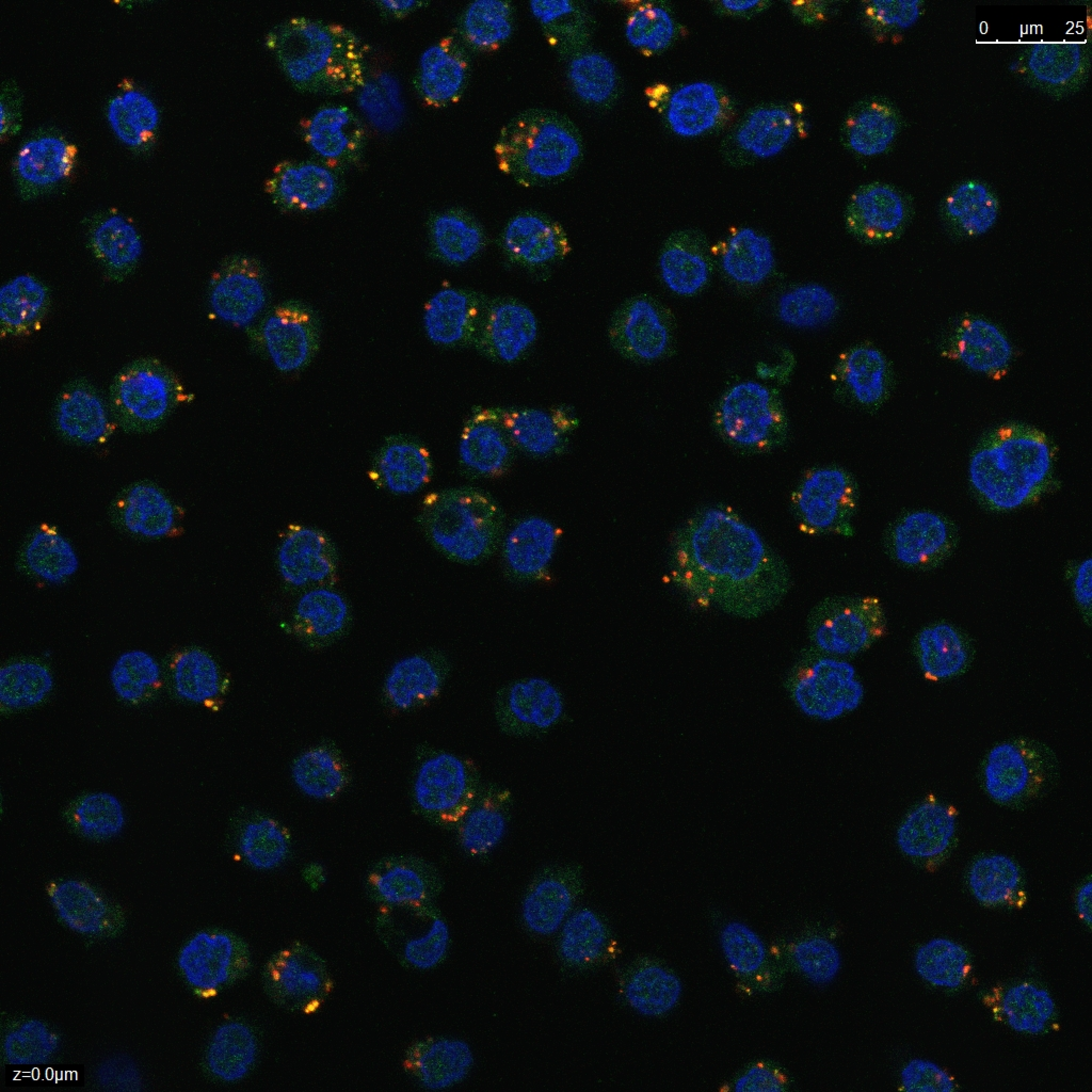
0 µm 25
z=0.0µm

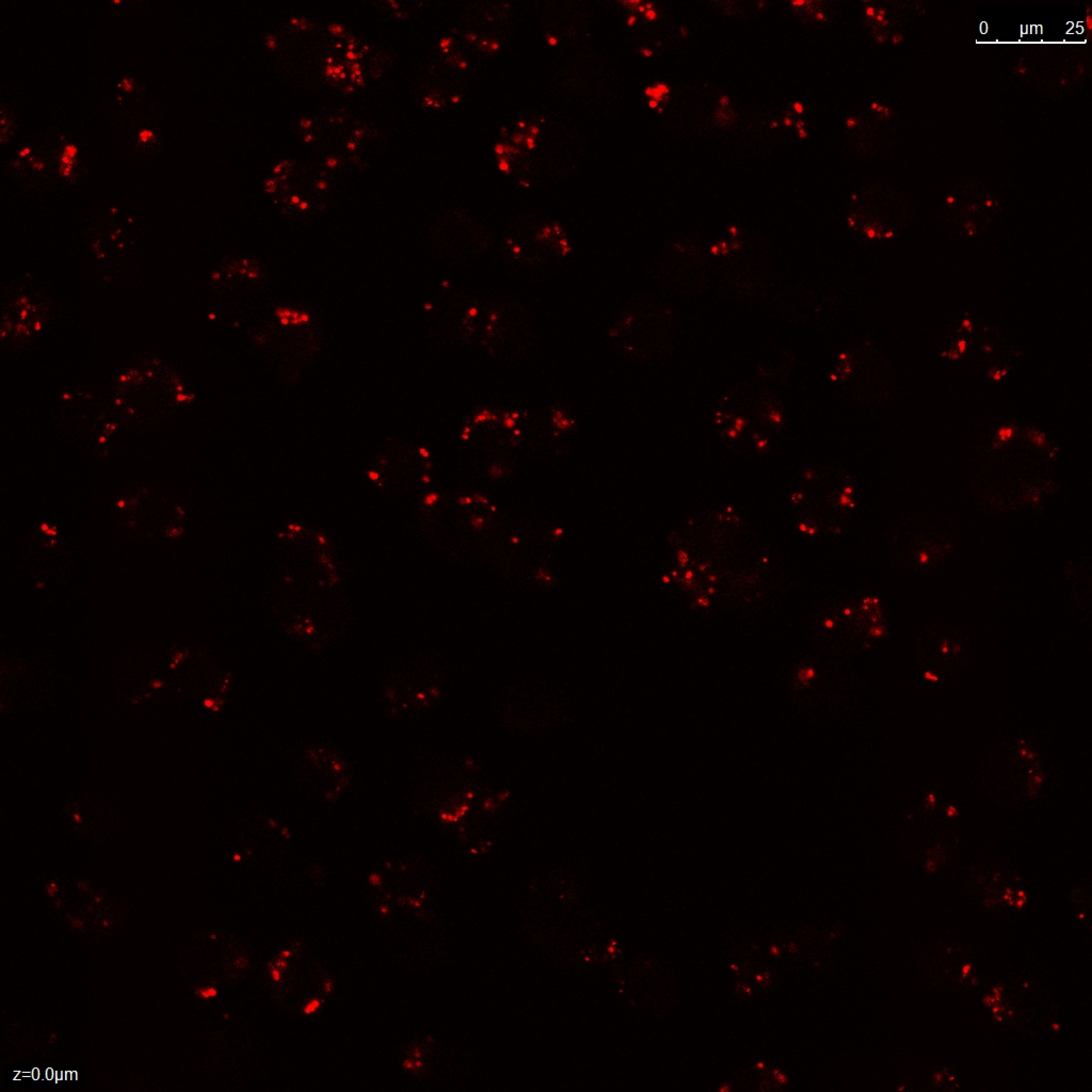
0 µm 25
z=0.0µm

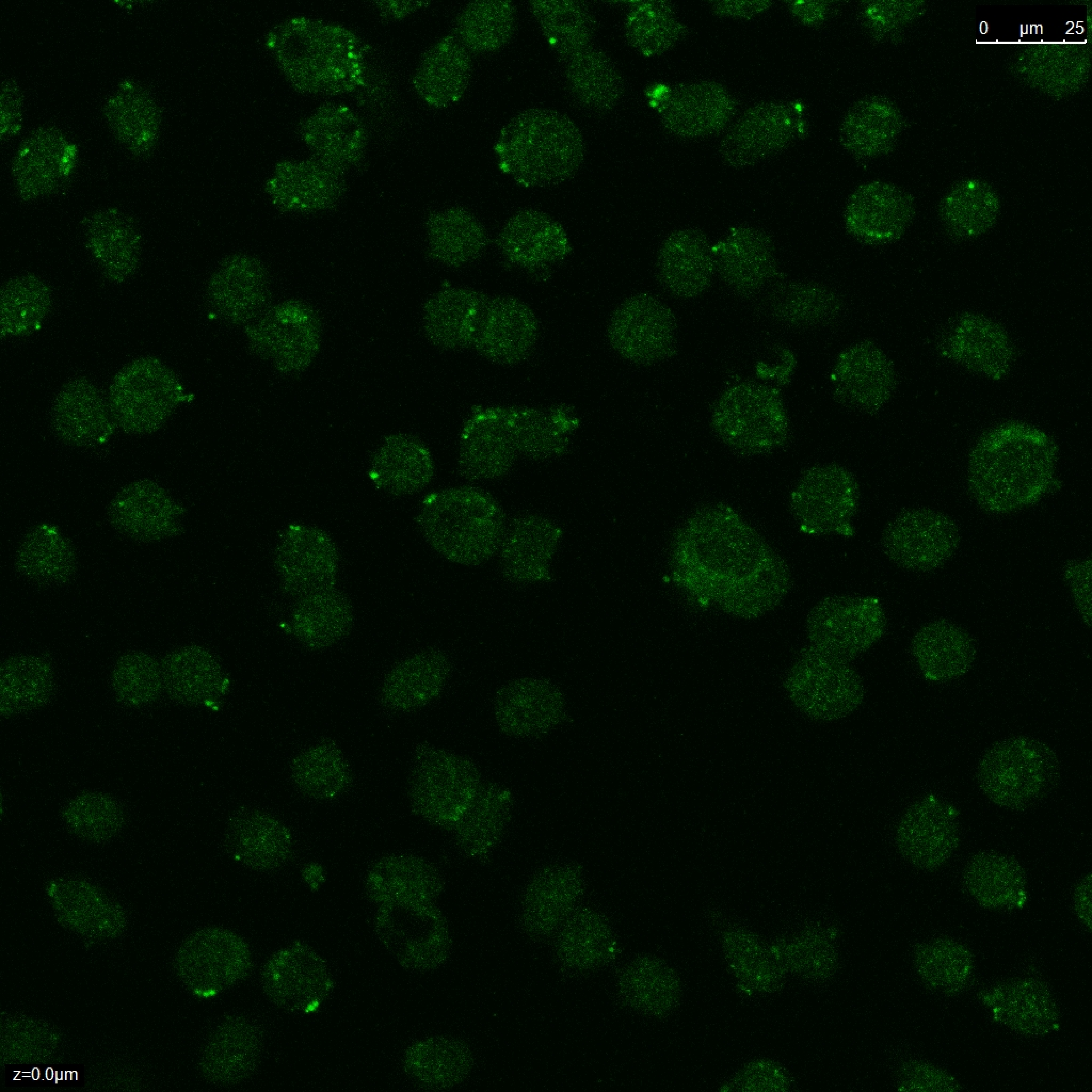
0 µm 25
z=0.0µm

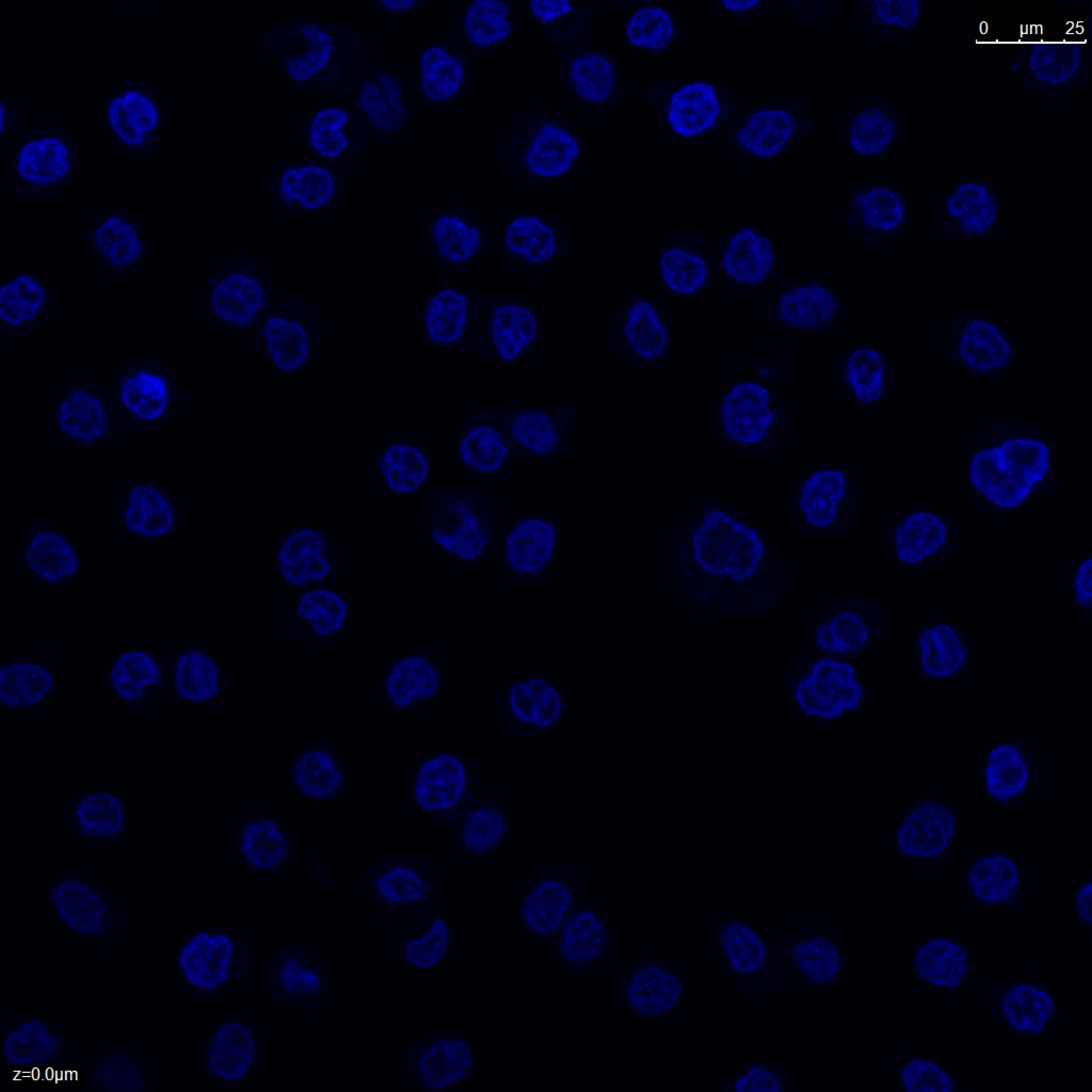
0 µm 25
z=0.0µm

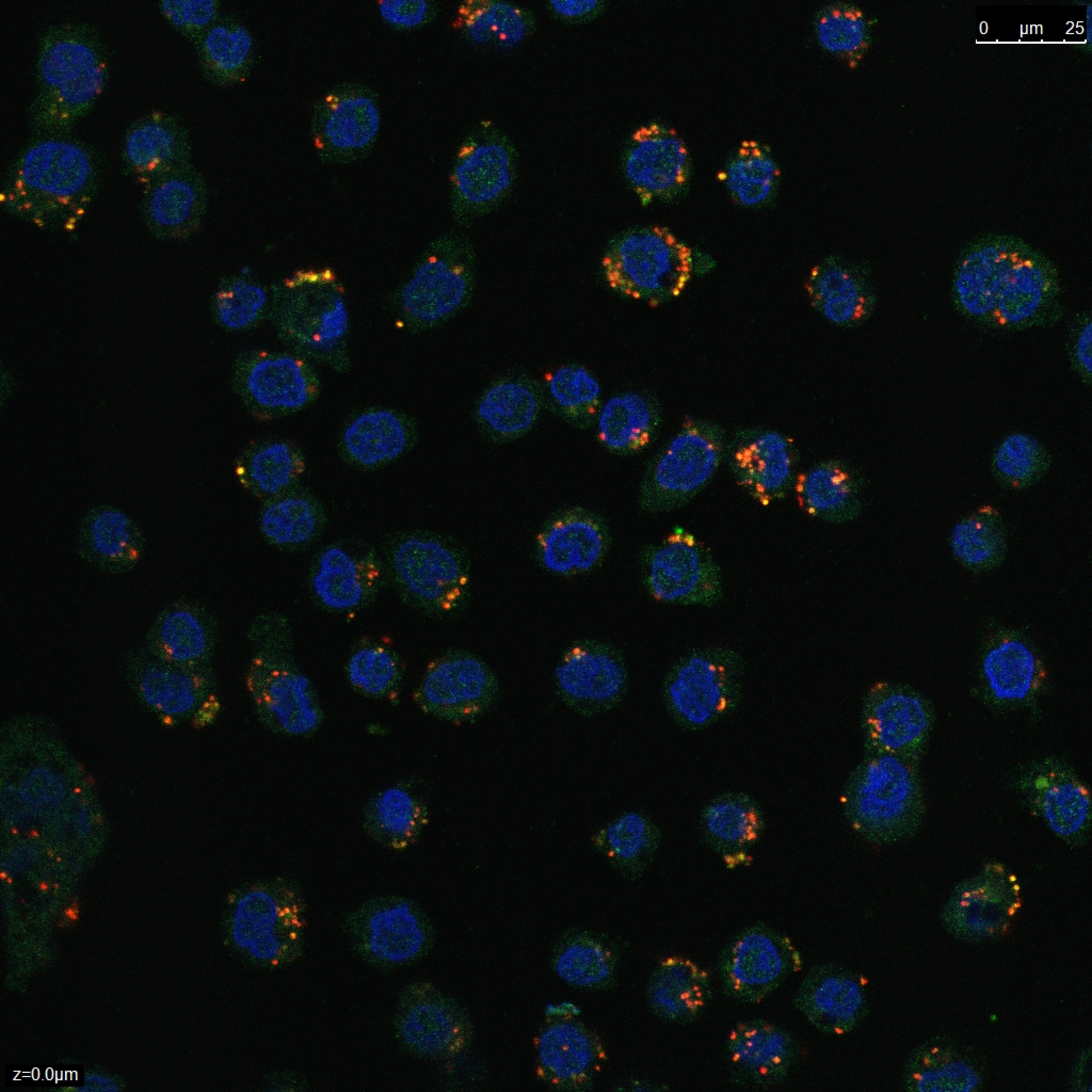
0 µm 25
z=0.0µm

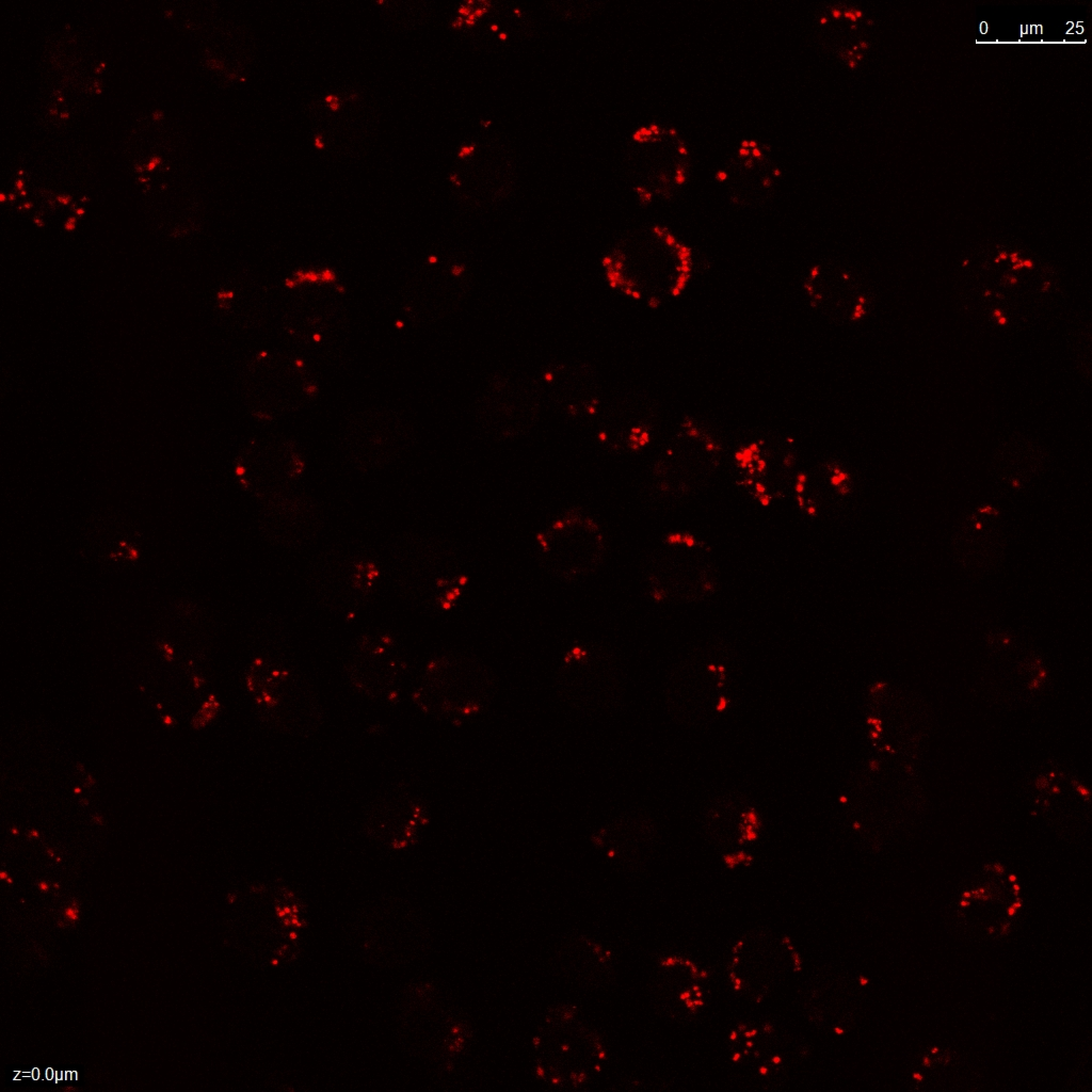
0 µm 25
z=0.0µm

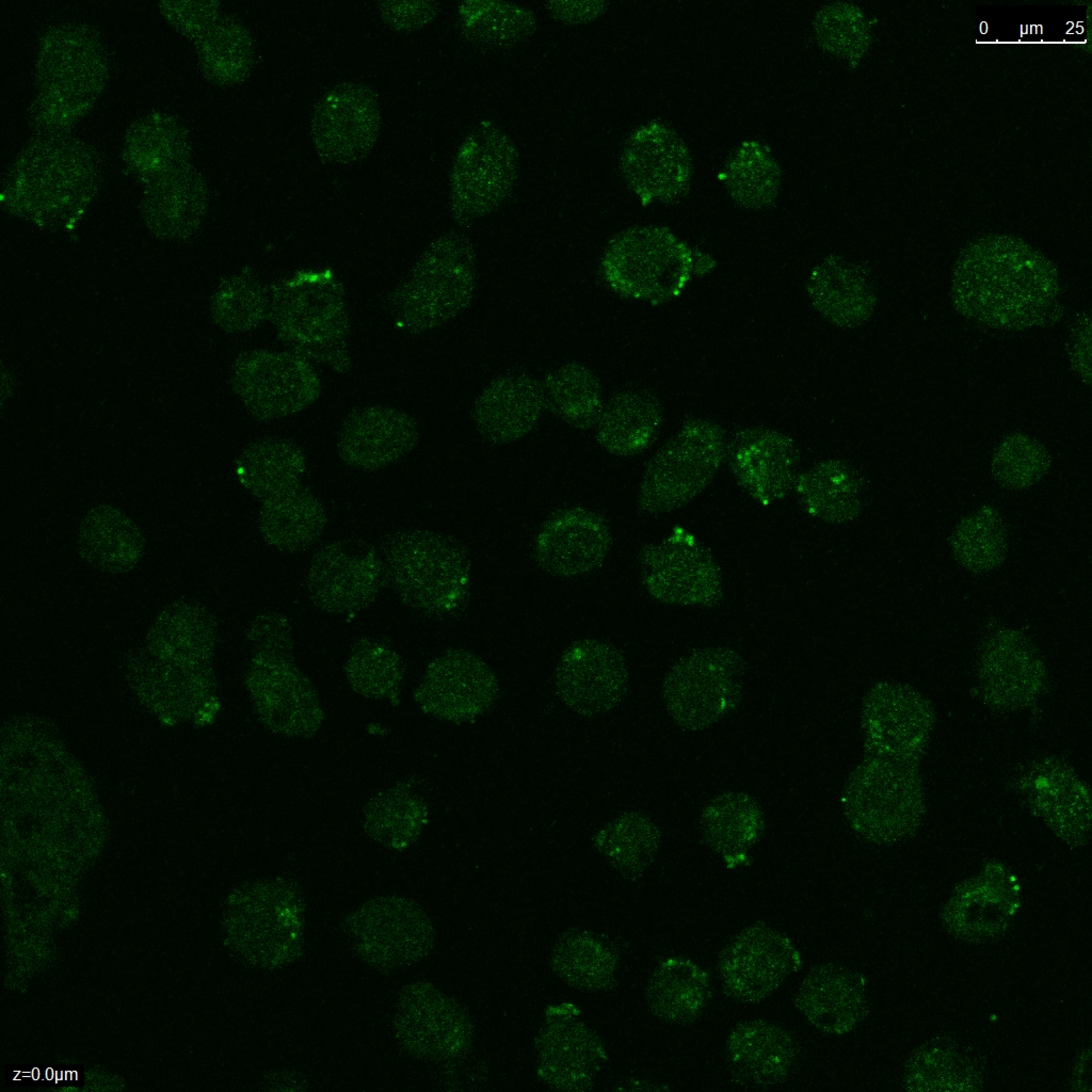
0 µm 25
z=0.0µm

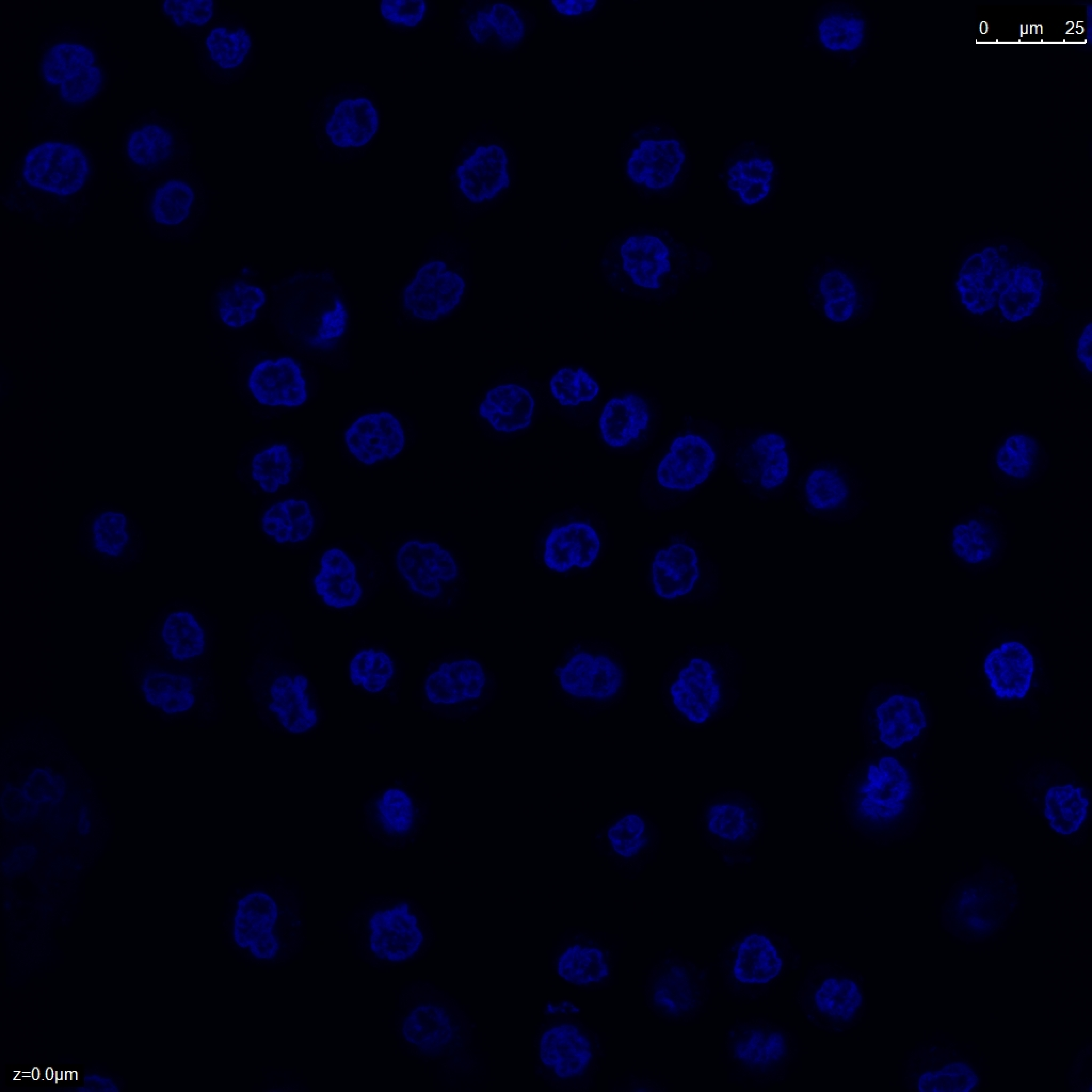

0 µm 25
z=0.0µm
