# Supplementary figures and images for: High expression of Helicobacter pylori VapD in both the intracellular environment and biopsies from gastric patients with severity
Source: PLoS One. 2020 Mar 12;15(3):e0230220. doi: 10.1371/journal.pone.0230220 (PMC7067408; doi:10.1371/journal.pone.0230220)

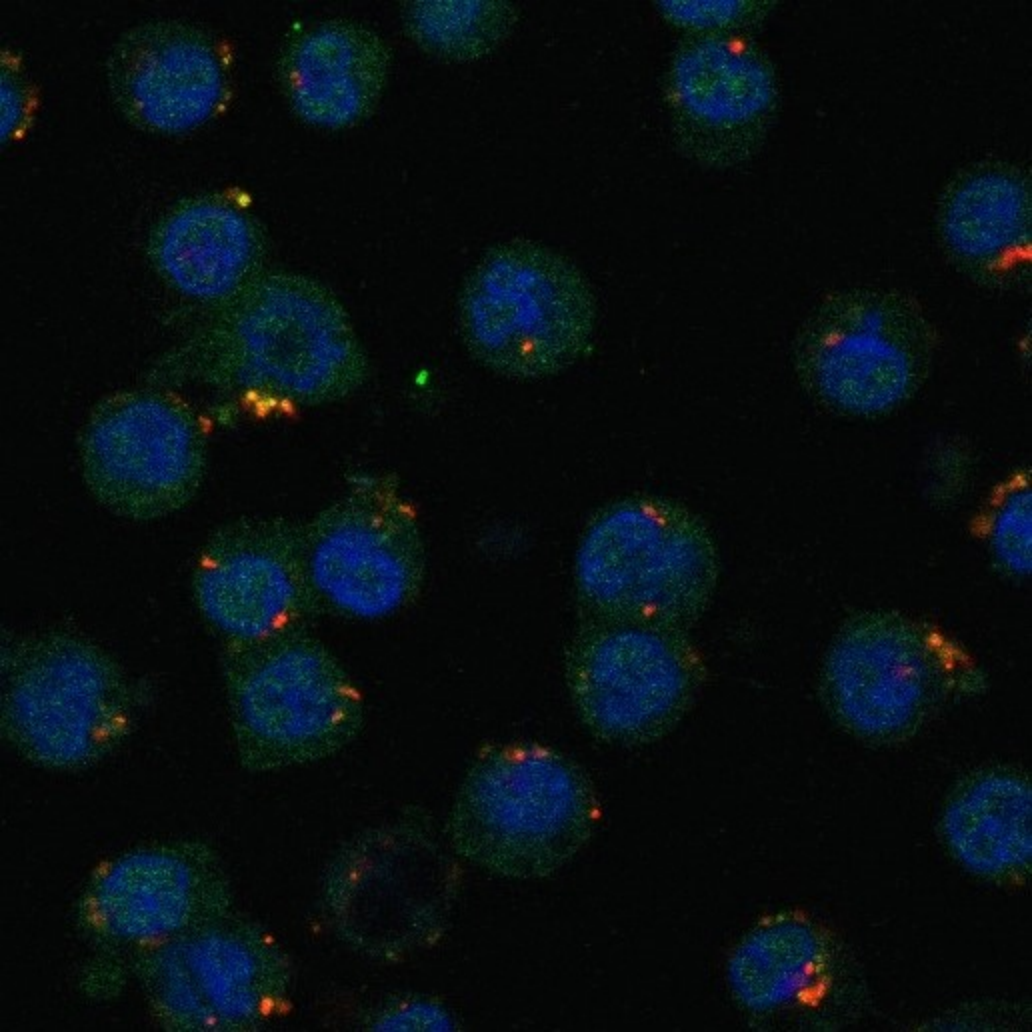

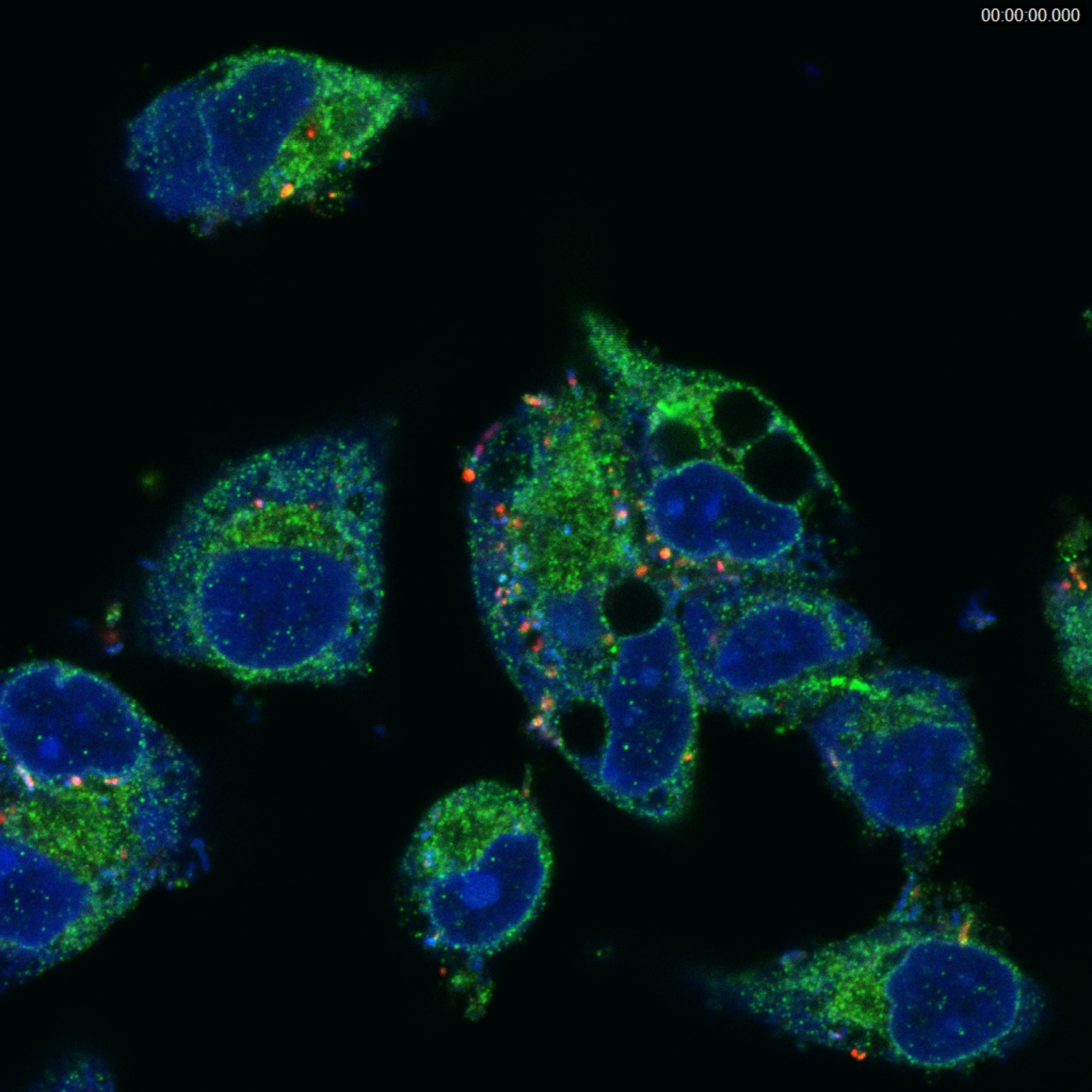

Supplement: S7 Raw images — (PDF) [file pone.0230220.s008.pdf]

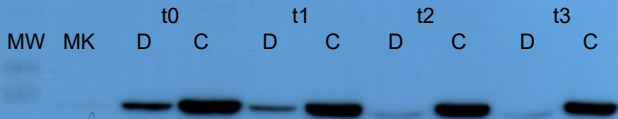

Exp 2

Gel 1

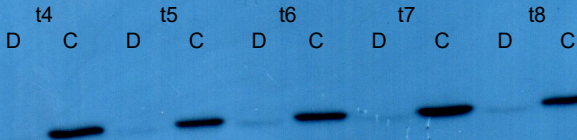

Exp 2 Gel 2

MW MK t0 t1 t2 t3  
D C D C D C D C

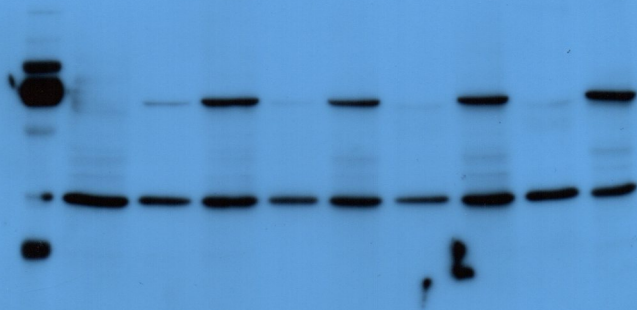

NAPDH Gel 1 & Gel 2

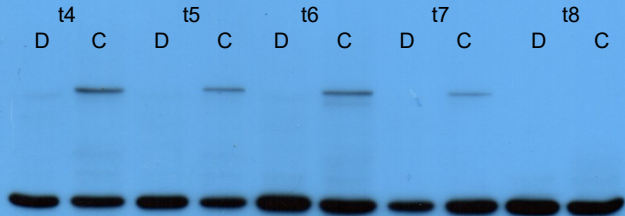

GAPDH Exp1

Gel 2

Supplement: S8 Raw images — (PDF) [file pone.0230220.s009.pdf]
