# Supplementary material for: High expression of Helicobacter pylori VapD in both the intracellular environment and biopsies from gastric patients with severity
Source: PLoS One. 2020 Mar 12;15(3):e0230220. doi: 10.1371/journal.pone.0230220 (PMC7067408; doi:10.1371/journal.pone.0230220)
Supplement: Attachment — Submitted filename: Response to Reviewers.doc [file pone.0230220.s010.doc]

1. We have eliminated from manuscript the phrase “data not shown” (line 309). It was substituted for the Figure 1, which show the results cited in the former paragraph.

Response to Reviewers

1. With respect to the article title

We are agree with the Review #1 about the article title, it is more convenient a title as: “ High expression of Helicobacter pylori VapD in both intracellular environment and biopsies from gastric patient with severity ”

2-6. We have reviewed the English grammar throughout the manuscript and the appropriate corrections have been made, which are indicated in red. Among them are those indicated by the reviewer on lines 30, 33, 71, 90, 92; which now correspond to lines 30, 32, 72, 91, 93 respectively.

On the other hand, we also change all assertive expressions used in the manuscript, instead of it, we "suggest" or "propose"
